# Pivotal role for typing of renal infiltrates: kidney localisation of a lymphoma presenting as pembrolizumab-associated nephritis. A lesson for the clinical nephrologist

**DOI:** 10.1007/s40620-025-02246-0

**Published:** 2025-05-12

**Authors:** Rayane Benyahia, Olivier Roques, Loïc Ysebaert, Yann Bergé, Julien Mazières, Charlotte Syrykh, Magali Colombat, Julie Belliere

**Affiliations:** 1 Department of Nephrology and Organ Transplantation, Referral Centre for Rare Kidney Diseases, University Hospital of Toulouse, Toulouse, France; 2 Department of Nephrology, Clinique Claude Bernard, Albi, France; 3 Department of Hematology, University Hospital of Toulouse, University Cancer Institute of Toulouse (Oncopole), Toulouse, France; 4 University Paul Sabatier-Toulouse 3, Toulouse, France; 5 Department of Medical Oncology, Clinique Claude Bernard, Albi, France; 6 Department of Pneumology, Larrey Hospital, University Hospital of Toulouse, Toulouse, France; 7 Department of Pathology, University Hospital of Toulouse, University Cancer Institute of Toulouse (Oncopole), Toulouse, France

**Keywords:** Tubulo-interstitial nephritis, Immune checkpoint inhibitors, Pembrolizumab; renal lymphoma, Small lymphocytic lymphoma, Rituximab

A 77-year-old woman with no significant past medical history except for type 2 diabetes, was diagnosed with a localised lung adenocarcinoma. After surgery, she received adjuvant treatment with three 28-day cycles of cisplatin (80 mg/m^2^ intravenous (IV) on day 1) and vinorelbine (30 mg/m^2^ IV on days 1 and 8). Metastatic progression compelled a second-line treatment with four 21-day cycles of carboplatin (AUC 5 IV on day 1), pemetrexed (500 mg/m^2^ IV on day 1) and the immune checkpoint inhibitor pembrolizumab (200 mg IV on day 1). Partial response then allowed for a maintenance therapy (21-day cycles) comprising only pemetrexed (500 mg/m^2^ IV on day 1) and pembrolizumab (200 mg IV on day 1). Because of haematological toxicity (anaemia) and infectious complications, pemetrexed was stopped after 5 cycles of maintenance therapy, whereas pembrolizumab was continued.

The patient was subsequently referred for acute kidney disease (AKD). Serum creatinine level (SCr) gradually increased from 0.7 mg/dL at baseline (before the first cycle of platinum), to 1.2 mg/dL when the patient was started on pembrolizumab as part of the second-line treatment, and then to 1.4 mg/dL once the immune checkpoint inhibitor became the exclusive antineoplastic therapy. Subsequently, SCr rose to 1.8 mg/dL in two months (Fig. 1), with near-normal urine protein-to-creatinine ratio (0.26 to 0.50 g/g), microalbuminuria (0.08 g/g), and low-grade sterile pyuria (47/µL, N < 20/µL), without haematuria. Routine blood screening for viral, dysimmune or complement-mediated nephropathies proved negative. Pembrolizumab as well as the proton pump inhibitor were discontinued in accordance with the current guidelines [1, 2], and a steroid regimen (starting at 1 mg/kg/day PO before tapering) was started immediately after performing a percutaneous kidney biopsy, as immune checkpoint inhibitor-mediated acute tubulo-interstitial nephritis (TIN) was strongly suspected.

**Fig. 1:**
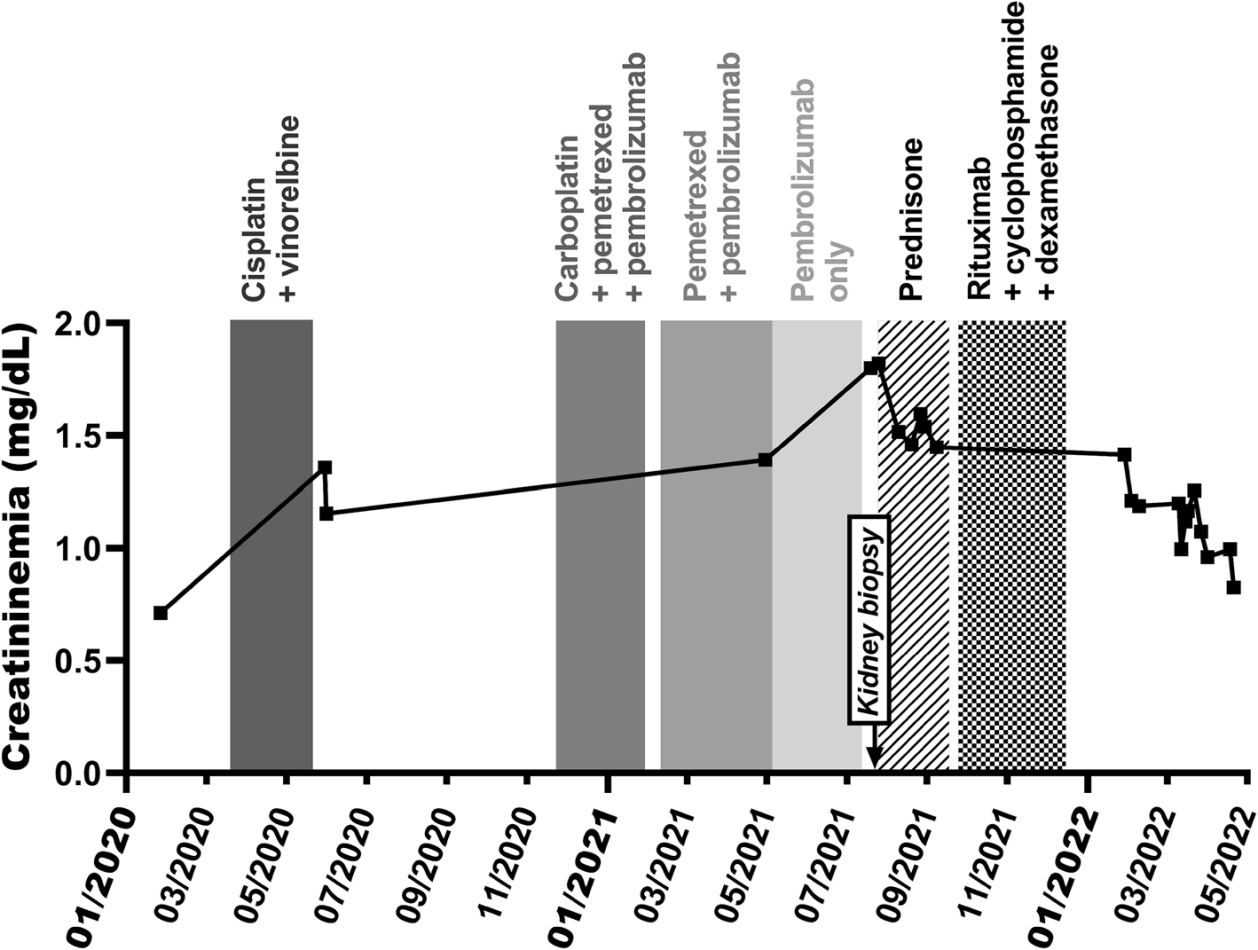
Evolution of kidney function over time. The graph depicts serum creatinine levels (in mg/dL, Y axis) over time (X-axis), with regard to anti-cancer protocols (shown as vertical grey rectangles). Conversion factor for creatininemia in mg/dL to µmol/L, × 88.4

Renal histology (Fig. 2) was characterized by tubular atrophy and interstitial fibrosis – elemental lesions that could account for immune checkpoint inhibitor-mediated TIN but also for chronic TIN caused by platinum-based drugs. Small interstitial infiltrates, however, argued for immune checkpoint inhibitor toxicity. In addition, 8 out of 26 glomeruli were globally sclerotic, a few showed hyalin deposits, and the rest appeared optically normal. Immunofluorescence (including kappa and lambda light chain immunostaining) was negative. However, immunohistochemistry revealed mainly CD20^+^ CD5^+^ interstitial infiltrates along with scarce, more scattered CD3^+^ lymphocytes, suggesting monoclonal infiltration. PCR performed on renal tissue confirmed monoclonal B-cell infiltration. EBER in situ hybridisation for Epstein-Barr virus was negative. This histology prompted us to ask for flow cytometric immunophenotyping of peripheral blood lymphocytes, which identified a subset of CD5^+^ CD19^+^ CD23^+^ CD79b^weak^ FMC7^weak^ surface lambda Ig^weak^ cells (i.e., Matutes score 5/5 cells) at 0.7 × 10^3^/µL. This high-count monoclonal lambda B-cell lymphocytosis (MBL) was not associated with anaemia nor thrombocytopenia, and radiological workup excluded lymphoma extension. Overall, a diagnosis of small lymphocytic lymphoma (SLL) restricted to kidneys was confirmed, instead of the initial suspicion of immune checkpoint inhibitor-mediated acute TIN.

**Fig. 2:**
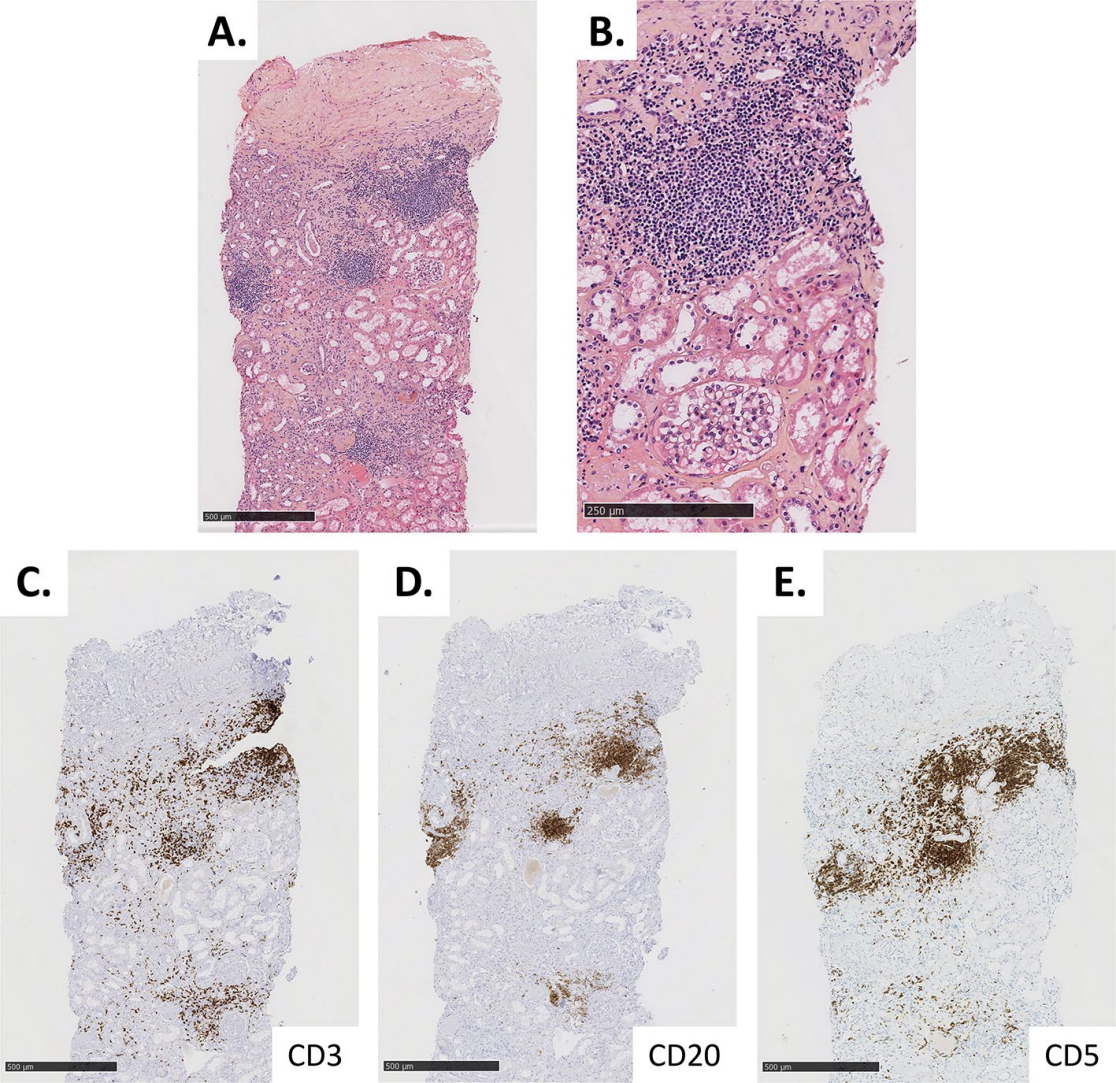
Kidney biopsy confirming the diagnosis of immune checkpoint inhibitor-associated, primary renal, small lymphocytic lymphoma. Panel **A** Hematoxylin and eosin (H&E) staining (5 × magnification), showing lymphoid interstitial infiltrates alongside interstitial fibrosis, tubular atrophy, and discrete to moderate atherosclerosis lesions. Panel **B** Close-up of the lymphoid infiltrates with H&E staining (10 × magnification). Panels **C**, **D**, **E** Immunohistochemical staining of CD3 (**B**), CD20 (**C**) and CD5 (**D**) (5 × magnification), showing that the above-mentioned lymphoid infiltration is mostly composed of CD20^+^ CD5^+^ B-cells (monoclonality proven by PCR)

From a therapeutic point of view, prednisone was stopped, and a new line of therapy including rituximab (375 mg/m^2^ IV on day 1), cyclophosphamide (300 mg PO on days 1 to 5) and dexamethasone (20 mg IV on day 1) was decided as a means to target the small lymphocytic lymphoma. This strategy proved effective with regard to kidney function after completing four cycles (21-day cycles) of immunochemotherapy (Fig. 1). Meanwhile, CT-scan follow-up demonstrated lung cancer progression, and the patient was switched to taxane-based palliative chemotherapy. Unfortunately, she died of post-COVID-19 complications.

## Lessons for the clinical nephrologist

Immune checkpoint inhibitors have emerged as groundbreaking therapies in the field of oncology, improving outcomes in numerous solid-organ and haematological cancers. While enhancing the antitumoural immune defense, anti-PD(L)1 or anti-CTLA4 agents may trigger immune-related adverse events (irAE) and result in increased morbidity-mortality through renal injury. Acute kidney injury (AKI) is the main type of renal immune-related adverse events, with a prevalence estimated at 2–5% [3]. Typical histology of immune checkpoint inhibitor-related AKI is characterised by acute TIN. The precise mechanisms underlying this renal toxicity remain unclear. Antigenic overlap between normal tubular cells and tumour cells could result in activation of self-reactive T-cell clones by the immune checkpoint inhibitor drug, leading to the T-cell infiltrates that are usually observed in immune checkpoint inhibitor-mediated TIN [3].

Suspicion of immune checkpoint inhibitor-mediated TIN usually arises from a (sub)acute increase of SCr associated with low-grade proteinuria and sterile pyuria, generally occurring 3 to 4 months after immune checkpoint inhibitor initiation. Whether kidney biopsy should be systematically performed whenever immune checkpoint inhibitor-mediated TIN is suspected continues to raise debate [4]. At present, histological confirmation (through kidney biopsy) is primarily limited to cases raising suspicion of a different diagnosis – e.g., high-grade proteinuria or corticosteroid resistance [1, 2]. Consequently, most studies reported low frequencies of patients on immune checkpoint inhibitor treatment experiencing AKI who underwent a kidney biopsy – ranging from 3.8 to 43% depending on screening criteria [5]. On one hand, assuming that most cases of immune checkpoint inhibitor-associated AKI are acute TIN, empirical prescription of corticosteroids would result in complete recovery in the majority of cases without risking complications of the kidney biopsy procedure. On the other hand, an incorrect diagnosis may expose the patient to both unnecessary steroid therapy (with its own adverse effects) and ill-advised immune checkpoint inhibitor discontinuation (possibly resulting in inferior cancer outcome). Moreover, as described in other forms of drug-induced TIN, clinical findings and laboratory tests seem to poorly predict the underlying kidney lesions of immune checkpoint inhibitor-related AKI [3]. Patients may also exhibit other patterns of lesions rather than classic TIN. Because there are currently no studies that assessed the risk–benefit ratio of various diagnostic strategies for immune checkpoint inhibitor-related kidney injury, real-life observations may help us define the best approach to screen for, and manage, this toxicity.

Through our case, we illustrated how kidney histology helped clinicians to diagnose a lymphoproliferative disorder in a setting that was highly suggestive of an immune checkpoint inhibitor-mediated TIN. Unveiling kidney involvement of lymphoproliferative disorders warrants fine-tuned characterisation of renal immune infiltrates. While small lymphocytic lymphoma is considered a monoclonal B-cell infiltration, TIN related to immune checkpoint inhibitor prescription is characterised by inflammatory infiltrates mostly composed of polyclonal CD3^+^ T-cells [3]. First-line treatments of these two entities differ significantly – rituximab-based immunochemotherapy for the former, a steroid regimen for the latter. The anti-CD20 monoclonal antibody rituximab has also been suggested as a safe and efficient treatment for other types of nephropathies that may be associated with (or triggered by) immune checkpoint inhibitor agents, such as pauci-immune vasculitis or membranous nephropathy [6]. Since clinical and biological data may be insufficient to differentiate immune checkpoint inhibitor-mediated TIN from other renal conditions, our case report advocates for the importance of kidney histology to explore immune checkpoint inhibitor-related kidney injuries whenever possible. Some immune checkpoint inhibitor-associated nephropathies may be incorrectly diagnosed as TIN if not for an appropriate diagnostic workup. For instance, in a French study that included 12 patients suspected of pembrolizumab-related kidney injury who underwent renal biopsy, only 4 were found to have acute TIN according to histology examination [3]. In addition, histology provides prognostic information that may be of importance for the patient’s care. All these statements fuel previous works questioning the relevance of current guidelines that restrict kidney biopsy indications [3, 4].

Additionally, we have hypothesised that pembrolizumab may have favoured this peculiar type of nephropathy. The most frequently reported haematological immune-related adverse events are thrombocytopenia, haemolysis, and aplastic anaemia. Albeit very rare, few cases of lymphoma and leukaemia occurring during immune checkpoint inhibitor therapies have been reported in the literature or in the WHO pharmacovigilance database (VigiBase) [7]. Supposing that our patient had had non-diagnosed chronic lymphocytic leukaemia or monoclonal lambda B-cell lymphocytosis prior to pembrolizumab therapy, recruitment of monoclonal B-cells into renal tissue could have been promoted by immune checkpoint inhibitor-mediated local inflammation. The hypothesis of malignant B-cell tropism for organs affected by inflammatory lesions has already been suggested in other organs [8]. Another explanation might be that selection and expansion of a pathogenic B-cell clone could be a direct consequence of immune checkpoint inhibitor-driven T-cell activation. In both scenarios, infusion of an anti-CD20 agent appears to be an appealing countermeasure. Rituximab is believed to suppress B-cell upregulation without impairing T-cell-mediated anti-tumour immunity [9]. In our case – the first immune checkpoint inhibitor-associated small lymphocytic lymphoma that has been reported to date in the literature – rituximab-based immunochemotherapy seemed to have improved the patient’s kidney function, achieving complete response according to recent recommendations (i.e., return of SCr to maximum 30 µmol/L above the baseline value) [5]. However, one cannot rule out that the steroids in this treatment protocol (dexamethasone) did not play a prominent role. In a broader sense, more experience is needed before drawing conclusions on this kind of immune-related adverse event management, including consideration of immune checkpoint inhibitor rechallenge.

Overall, this clinical vignette emphasises three points. Firstly, kidney biopsy remains essential when dealing with suspected immune checkpoint inhibitor-mediated acute TIN, because this therapeutic class is also known for triggering other types of nephropathies that may require safe non-steroid prescriptions (e.g., anti-CD20 antibodies). Secondly, kidney histology in this setting should always incorporate immunophenotyping, in order not to miss monoclonal lymphoproliferative disorders that could be mistaken for common polymorphous inflammatory infiltrates. Lastly, this case raises awareness of malignant haemopathies as either potentially underestimated immune-related adverse events or differential diagnoses of the same.

## Data Availability

Data sharing is not applicable to this article as no datasets were generated or analysed during the current study.
